# Investigation of diets associated with dilated cardiomyopathy in dogs using foodomics analysis

**DOI:** 10.1038/s41598-021-94464-2

**Published:** 2021-08-05

**Authors:** Caren E. Smith, Laurence D. Parnell, Chao-Qiang Lai, John E. Rush, Lisa M. Freeman

**Affiliations:** 1 Nutrition and Genomics Laboratory, Jean Mayer USDA Human Nutrition Research Center on Aging at Tufts University, Boston, MA USA; 2 USDA Agricultural Research Service, Nutrition and Genomics Laboratory, Jean Mayer USDA Human Nutrition Research Center on Aging at Tufts University, Boston, MA USA; 3 Department of Clinical Sciences, Cummings School of Veterinary Medicine, Tufts University, North Grafton, MA USA

**Keywords:** Biochemistry, Cardiology

## Abstract

Dilated cardiomyopathy (DCM) is a disease of the heart muscle that affects both humans and dogs. Certain canine diets have been associated with DCM, but the diet-disease link is unexplained, and novel methods are needed to elucidate mechanisms. We conducted metabolomic profiling of 9 diets associated with canine DCM, containing ≥ 3 pulses, potatoes, or sweet potatoes as main ingredients, and in the top 16 dog diet brands most frequently associated with canine DCM cases reported to the FDA (3P/FDA diets), and 9 non-3P/FDA diets. We identified 88 named biochemical compounds that were higher in 3P/FDA diets and 23 named compounds that were lower in 3P/FDA diets. Amino acids, amino acid-derived compounds, and xenobiotics/plant compounds were the largest categories of biochemicals that were higher in 3P/FDA diets. Random forest analyses identified the top 30 compounds that distinguished the two diet groups with 100% predictive accuracy. Four diet ingredients distinguished the two diet groups (peas, lentils, chicken/turkey, and rice). Of these ingredients, peas showed the greatest association with higher concentrations of compounds in 3P/FDA diets. Moreover, the current foodomics analyses highlight relationships between diet and DCM in dogs that can identify possible etiologies for understanding diet-disease relationships in dogs and humans.

## Introduction

Dilated cardiomyopathy (DCM) is a common, progressive, and “largely irreversible” heart disease affecting humans^1^. Dilated cardiomyopathy is associated with left ventricular dilation and systolic dysfunction, and commonly leads to congestive heart failure or sudden death^1,2^. Dilated cardiomyopathy can result from both genetic and environmental causes. Genetic mutations contribute to DCM in humans but known mutations are identified in only 25–40% of those with familial DCM, reflecting substantial gaps in understanding of the causes of DCM^3^. In addition, the natural history of DCM in humans is highly variable suggesting that environmental factors may affect disease progression even in DCM with a genetic basis.

This disease does not only occur in humans; DCM is the second most common heart disease affecting pet dogs, with prevalence over 50% in some breeds, such as the Doberman Pinscher^4–6^. DCM in dogs, as in humans, is a serious disease leading to congestive heart failure or sudden death, with survival times typically less than one year after the onset of heart failure and significant echocardiographic improvement unlikely^5^. Several genetic mutations also have been associated with DCM in dogs but most dogs with canine DCM do not have a genetic mutation identified^7–11^. Even in the Doberman Pinscher, in which two different mutations associated with DCM have been identified, some dogs with DCM have a single mutation, some have both mutations, and some have neither^7–9^. This variable genetic background and a highly variable disease progression, even in dogs with the same mutation, also support a role for environmental factors in canine DCM.

In addition to genetic causes, DCM can also occur secondary to environmental causes such as direct toxins (e.g., alcohol, chemotherapeutic agents, antibiotics, heavy metals), infectious agents, and nutritional deficiencies^12–14^. Deficiencies of a variety of nutrients, such as thiamine, magnesium, choline, vitamin E and selenium, have been associated with DCM in humans or animal models^12,15–17^. Deficiencies of two amino acid or amino acid-related compounds also can cause a nutritional DCM. Taurine deficiency has been investigated extensively in cats with DCM, where it is known to cause DCM that is reversible with taurine supplementation^18,19^. In dogs, the role of taurine in DCM is less clear but has been associated with DCM, particularly in certain breeds^20–35^. Deficiency of L-carnitine, an amino acid-derived molecule, also has been implicated in DCM in dogs and humans^36,37^. Carnitine belongs to a chemical category of trimethylated quaternary amines and imines called “betaines,” which have been identified in many foods and linked to human health^38,39^.

Recent focus on the role of diet in DCM in dogs stems from veterinary reports and Food and Drug Administration (FDA) investigation of a potential link between certain diets and DCM in dogs^34,40–46^. As of September, 2020, > 1100 dogs with DCM had been reported to the FDA^47^. One of the unique characteristics about the dogs with diet-associated DCM is their outcome. While DCM in dogs is usually a progressive disease with short survival times, multiple studies have shown that dogs with diet-associated DCM have significant echocardiographic improvement and longer survival time after diet change and medical treatment^34,40,44–46^. The diets reported to be associated with DCM often are marketed as “grain-free” and often contain certain ingredients that became part of commercial foods relatively recently (e.g., pulses, potatoes, and sweet potatoes) and lack others (such as rice or corn). Most of the ingredients that are included in the associated diets are also found in human diets, but dogs often eat them in even higher quantities because most dogs eat a single commercial pet food, rather than a variable mixture of multiple foods as humans do. However, intake of certain pet food ingredients such as pulses and tubers vary widely among human populations, and in some groups represent staple foods that provide substantial sources of protein and energy. Despite ongoing research efforts, understanding of whether diet may be involved in the observed DCM in dogs remains unclear. Standard nutritional analyses of the associated diets have failed to identify a causative factor and so other, more novel approaches are needed.

One novel approach for studying relationships between diet and diseases is metabolomics, which measures small molecules that can identify biomarkers of disease or diet, as well as to help delineate mechanistic pathways^48,49^. While metabolites are typically assessed in plasma or urine, this method can also be used to compare the same biochemical compounds in foods^50^, where it has been referred to as “foodomics”^51^. Identifying the biochemical distinguishers of diets associated with DCM in dogs and more traditional dog foods could reveal possible biochemical compounds in food that are contributing to DCM in pet dogs, and also may help to identify food ingredients and biochemical compounds that could play a role in human DCM as well. The study’s primary objective, therefore, was to apply a metabolomics approach to identify biochemical compounds that differ between commercial dog foods that have been associated with canine DCM and in more traditional commercial dog foods.

## Results

### Biochemical compounds differ in two diet groups

A total of 830 biochemical compounds (665 of known identity and 165 of unknown identity) were measured and compared in two diet groups. One group (called 3P/FDA; n = 9) consisted of diets clinically associated with DCM in dogs, from the top 16 dog food brands fed to dogs with DCM reported to the FDA, and contained  ≥ 3 pulses, potatoes, or sweet potatoes in the top 20 ingredients^43^. The comparison group (non-3P/FDA; n = 9) were diets not associated clinically with DCM in dogs and did not meet the other criteria for the 3P/FDA diets.

**Figure 1:**
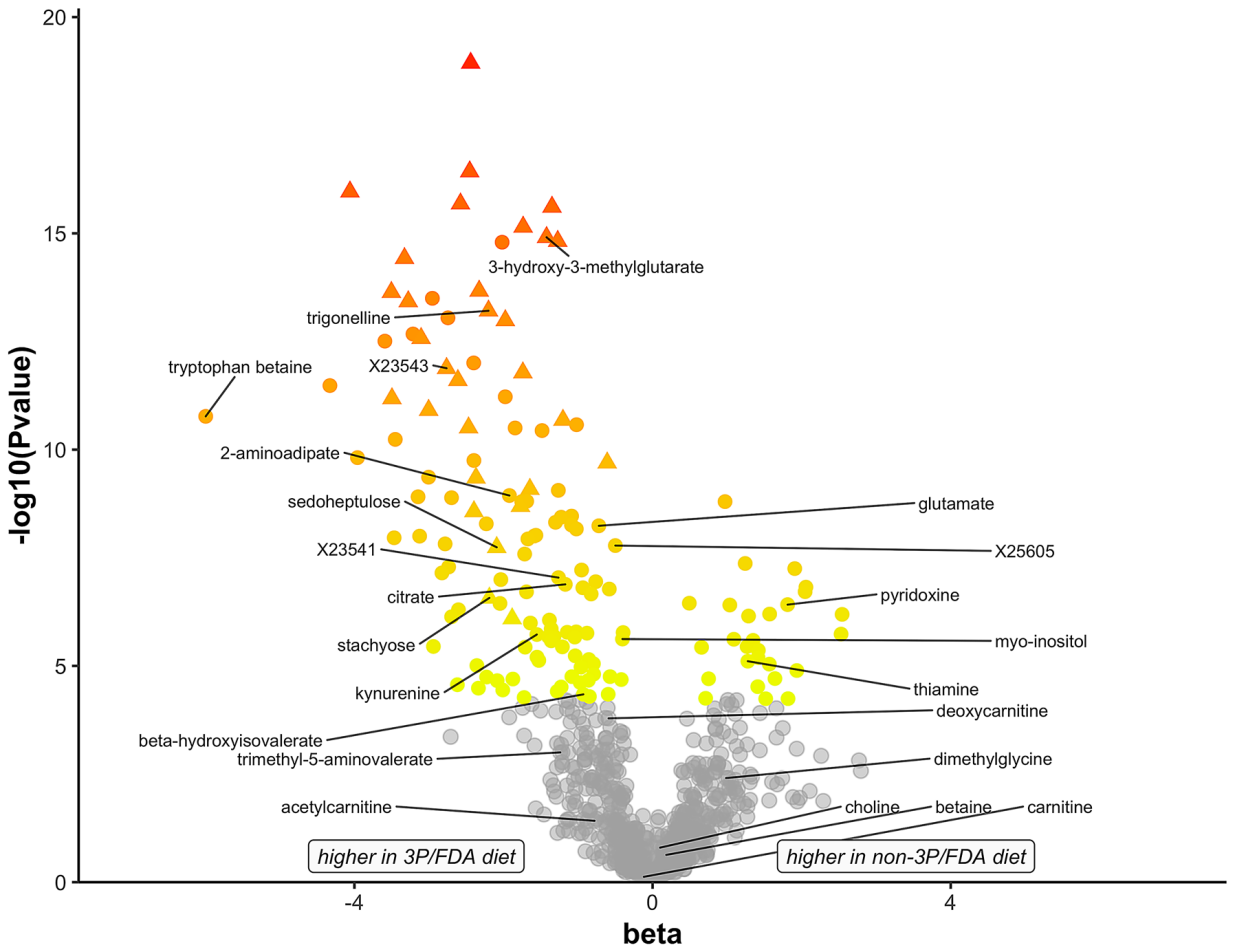
Illustration of 830 biochemical compounds that were significantly different between diet groups. Diets were assigned to one of two groups: (1) associated with clinical cases of dilated cardiomyopathy (DCM) in dogs; containing ≥ 3 pulses, potatoes, or sweet potatoes in the top 20 ingredients; and in the top 16 dog food brands named most frequently in DCM cases reported to the FDA (3P/FDA) or (2) not meeting the above criteria (non-3P/FDA). The beta values, where a negative value denotes higher levels in the 3P/FDA diet group, are plotted against the negative of log_10_(*P* value). Compounds plotted in gray have *P* values above the cutoff of 5.80E−05, and are considered as not statistically significantly different between diet groups. Triangles indicate compounds that distinguish the two diet groups based on random forest analysis. Those compounds featured in this manuscript are labeled.

Regression coefficients and the log_10_ *P* values from the 3P/FDA and non-3P/FDA diet comparison are plotted to show the extent of chemical differences between the diet groups (Fig. 1). Named and unnamed compounds with negative beta coefficients (n = 122, 81.9%) showed higher concentrations in the 3P/FDA diets, while named and unnamed compounds with positive beta coefficients were lower in 3P/FDA diets (n = 27, 18.1%; Supplemental Table 1).

**Table 1:** Biochemical compounds that were higher in the 3P/FDA diet group compared to the non-3P/FDA diet group^43^.

Chemical or functional class	Compound	Fold	Beta	SE	P
**Amino Acids**		Diff			
Alanine and aspartate metabolism	Asparagine	2.88	− 1.024	0.177	1.63E−06
Glutamate metabolism	Glutamate	1.96	− 0.721	0.094	5.76E−09
Glutathione metabolism	Glutathione, oxidized (GSSG)^1^	11.20	− 2.612	0.246	2.49E−12
Leucine, isoleucine and valine	3-Methylglutaconate	3.04	− 1.086	0.138	3.43E−09
Leucine, isoleucine and valine	Methylsuccinate	3.23	− 0.970	0.199	2.42E−05
Leucine, isoleucine and valine	Beta-hydroxyisovalerate	2.49	− 0.929	0.199	4.54E−05
Lysine metabolism	N6,N6-dimethyllysine^1^	4.26	− 1.646	0.196	8.19E−10
Lysine metabolism	2-Aminoadipate	6.72	− 1.920	0.232	1.15E−09
Lysine metabolism	N6-methyllysine	5.55	− 1.639	0.276	1.02E−06L
Lysine metabolism	N,N-dimethyl-5-aminovalerate	4.91	− 1.359	0.241	2.63E−06
Lysine metabolism	6-Oxopiperidine-2-carboxylate	2.48	− 0.855	0.162	7.13E−06
Lysine metabolism	Hydroxy-N6,N6,N6-trimethyllysine^2^	2.62	− 0.847	0.183	5.11E−05
Lysine metabolism	Pipecolate	12.59	− 1.723	0.373	5.38E−05
Methionine, cysteine, taurine	Methionine sulfone	8.48	− 1.976	0.193	5.99E−12
Polyamine metabolism	4-Acetamidobutanoate	3.68	− 1.264	0.151	8.73E−10
Tryptophan metabolism	Tryptophan betaine^2^	525.21	− 5.995	0.608	1.70E−11
Tryptophan metabolism	Kynurenine	5.66	− 1.553	0.271	1.88E−06
Tyrosine metabolism	Gentisate	4.56	− 1.527	0.289	7.46E−06
Urea cycle; arginine, proline	Dimethylarginine (ADMA + SDMA)^1^	6.28	− 1.975	0.165	1.03E−13
Urea cycle; arginine, proline	N-monomethylarginine^1^	4.88	− 1.740	0.161	1.66E−12
Urea cycle; arginine, proline	Homoarginine	14.02	− 4.329	0.412	3.30E−12
Urea cycle; arginine, proline	Homocitrulline	7.40	− 2.397	0.267	1.78E−10
Urea cycle; arginine, proline	Argininate	1.81	− 0.570	0.114	1.77E−05
Urea cycle; arginine, proline	N-delta-acetylornithine	17.60	− 2.010	0.423	3.60E−05
**Xenobiotics/Plant compounds**					
	Afromosin	47.15	− 2.358	0.454	9.81E−06
	Formononetin	40.18	− 3.146	0.381	1.23E−09
	Fucitol	6.43	− 1.586	0.211	9.89E−09
	Galactarate (mucic acid)	10.32	− 2.231	0.288	5.18E−09
	Gentisic acid-5-glucoside	7.04	− 2.604	0.421	5.03E−07
	Histidine betaine (hercynine)^2^	8.30	− 2.400	0.218	9.88E−13
	Hydroquinone beta-D-glucopyranoside	26.23	− 2.954	0.237	3.17E−14
	3-Hydroxybenzoate	38.43	− 2.696	0.445	7.31E−07
	4-Hydroxybenzoate	1.72	− 0.595	0.127	4.53E−05
	Kaempferol 3-O-glucoside/galactoside	27.90	− 2.084	0.424	2.19E−05
	Maltol	2.64	− 1.145	0.198	1.67E−06
	Nicotianamine^1^	15.61	− 3.278	0.265	3.75E−14
	Pheophorbide A	23.79	− 2.227	0.447	1.81E−05
	Protocatechuic acid-3-glucoside	4.20	− 1.302	0.167	4.79E−09
	Salicylate	13.53	− 1.874	0.379	2.01E−05
	Salicylate-glucoside	44.15	− 3.125	0.416	9.99E−09
	Soyasaponin I	5.41	− 2.736	0.394	5.15E−08
	Sulfate	1.47	− 0.395	0.068	1.69E−06
	Tartarate	45.77	− 3.959	0.439	1.53E−10
	Trigonelline^2^	7.76	− 2.201	0.181	6.16E−14

*P* value of 0.05/830 = 0.00006 was statistically significant. P reflects difference in concentration between 3P/FDA and non-3P/FDA diet groups.

Amino acids and xenobiotics/plant compounds are included here. In all, 122 named and unnamed biochemical compounds were significantly higher in the 3P/FDA diet group compared to the non-3P/FDA diet group. Complete list of compounds in Supplemental Table 1.

^1^Member of 30 compound set determined by random forest analyses to distinguish the 3P/FDA and non-3P/FDA diet groups.

^2^Betainized compound.

Of the compounds that were significantly higher in the 3P/FDA diet group, the largest categories of named compounds were amino acid related (n = 24), and those classed as xenobiotics/plant compounds (n = 20; Table 1). Other categories of compounds that were higher in the 3P/FDA diet group included lipids (n = 18), carbohydrates (n = 7), energy metabolism (n = 6), cofactors and vitamins (n = 5), nucleotides (n = 5), and peptides (n = 3). A large proportion of compounds that were higher in the 3P/FDA diet group were unnamed (n = 34; Supplemental Table 1). Three of these unnamed compounds were detectable only in the 3P/FDA diet group (X-23541, X-23534, and X-25605).

Of the compounds that were significantly lower in the 3P/FDA diet group, the largest category of named compounds was cofactors and vitamins (n = 8). Other categories that were lower included xenobiotics/plant compounds (n = 6), lipids (n = 5), amino acids (n = 3), and carbohydrates (n = 1). There were 4 unnamed compounds that were lower in 3P/FDA diet group.

Using random forest analysis, a set of 30 biochemical compounds that distinguished the 3P/FDA and non-3P/FDA diet groups was identified, yielding a predictive accuracy of 100%. Predictive ranks (1 to 30) reflect the biochemical importance of each of these 30 compounds (Supplemental Table 1). Included among these distinguishing compounds are amino acids, cofactors and vitamins, peptides, lipids, nucleotides, carbohydrates (stachyose and sedoheptulose), and 14 unnamed compounds. As plotted in Fig. 1, all 30 compounds distinguishing the two diet groups were higher in the 3P/FDA diet group.

### Vitamins were lower in 3P/FDA diets

Seven of the 8 vitamins that were significantly lower in 3P/FDA diets were B vitamins: pyridoxine (vitamin B6), thiamine (vitamin B1), folate (vitamin B9), pantothenate (vitamin B5), and riboflavin (vitamin B2) were lower in 3P/FDA diets (Table 2).

**Table 2:** Biochemical compounds that were lower in the 3P/FDA diet group compared to the non-3P/FDA diet group.

Chemical or functional class	Compound	Fold	Beta	SE	*P* value
**Cofactors and vitamins**		Diff			
Folate metabolism	Folate	0.15	1.419	0.260	4.36E−06
Nicotinate and nicotinamide metabolism	Nicotinate	0.20	1.411	0.293	3.02E−05
Pantothenate and CoA metabolism	Pantothenate	0.49	0.658	0.119	3.71E−06
Riboflavin metabolism	Riboflavin	0.14	1.907	0.276	5.62E−08
Thiamine metabolism	Thiamin	0.27	1.278	0.243	7.78E−06
Tocopherol metabolism	Gamma-tocotrienol	0.11	1.935	0.380	1.28E−05
Vitamin B6 metabolism	Pyridoxal	0.59	0.493	0.078	3.55E−07
Vitamin B6 metabolism	Pyridoxine	0.20	1.812	0.289	3.83E−07
**Xenobiotics/plant compounds**					
	Deoxymugineic acid	0.28	1.091	0.193	2.42E−06
	Feruloylquinate (1)	0.11	2.057	0.312	1.51E−07
	Feruloylquinate (3)	0.22	1.349	0.239	2.57E−06
	Feruloylquinate (4)	0.22	1.415	0.266	6.42E−06
	1,1-Kestotetraose	0.06	2.529	0.440	1.84E−06
	2-Oxindole-3-acetate	0.04	2.543	0.417	6.43E−07
**Lipids**					
Galactosyl glycerolipids	1,2-dilinoleoyl-galactosylglycerol (18:2/18:2)	0.13	1.567	0.301	9.10E−06
Galactosyl glycerolipids	1-palmitoyl-galactosylglycerol (16:0)	0.12	1.818	0.396	5.71E−05
Galactosyl glycerolipids	1-linoleoyl-galactosylglycerol (18:2)	0.20	1.518	0.330	5.74E−05
Sphingolipid synthesis	Phytosphingosine	0.26	1.243	0.177	4.28E−08
Sphingolipid synthesis	Sphingadienine	0.16	2.047	0.315	1.92E−07
**Amino acids**					
Tryptophan metabolism	Indoleacetate	0.29	1.288	0.212	7.03E−07
Tryptophan metabolism	Serotonin	0.20	1.642	0.331	1.96E−05
Tyrosine metabolism	1-Carboxyethyltyrosine	0.49	0.712	0.155	5.61E−05
**Carbohydrate**					
Glycolysis, gluconeogenesis, and pyruvate metabolism	Glucose	0.30	1.268	0.230	3.57E−06

*P* value of 0.05/830 = 0.00006 was statistically significant^.^ P reflects difference in concentration between 3P/FDA and non-3P/FDA diet groups.

Complete list of compounds in Supplemental Table 1.

### Amino acids, amino acid derivatives, and plant-derived compounds differ by diet group

As indicated above, a number of amino acids and amino acid-related compounds differed significantly between 3P/FDA and non-3P/FDA diets. Tryptophan betaine (525.21 fold), glutamate (1.96 fold), kynurenine (5.66 fold), 2-aminoadipate (6.72 fold; also called 2-aminoadipic acid), pipecolate (12.59 fold), N6,N6-dimethyllysine (4.26 fold), beta-hydroxyisovalerate (2.49 fold; also called 3-hydroxyisovaleric acid), and homoarginine (14.02 fold) were among those that were higher in the 3P/FDA diet group (Table 1). Taurine was not significantly different between the diet groups.

Acetyl-D,L carnitine (the D isomer of carnitine; *P* = 0.038), gamma butyrobetaine (also called deoxycarnitine; *P* = 0.0002), 5-aminovaleric acid betaine (also called *N,N,N*-trimethyl-5-aminovalerate; *P* = 0.001), and dimethyl glycine (*P* = 0.004) were higher with nominal significance (*P* < 0.05) in the 3P/FDA diet group (Supplemental Table 1). The plant-derived betaine compounds, trigonelline (N-methylnicotinic acid) and histidine betaine were significantly higher in the 3P/FDA diets (7.76 fold and 8.30 fold, respectively; Table 1). Carnitine did not differ significantly between the two diet groups.

### Other metabolites of interest

Three additional compounds were also significantly higher in the 3P/FDA diet group: 3-hydroxymethylglutaric acid, myo-inositol, and citrate (Supplemental Table 1).

### Ingredients are associated with biochemical compound concentrations

Based on biochemical compounds that differed between 3P/FDA and non-3P/FDA diets and differences in some ingredients across diet groups, we reasoned that ingredients could be contributing to the biochemical differences. To investigate ingredient-compound relationships, we first identified “distinguishing ingredients” that differed by five or more dog food products when compared across 3P/FDA and non-3P/FDA diet groups. These four ingredients were as follows: (1) peas (present in 9 3P/FDA diets and 4 non-3P/FDA diets), (2) lentils (present in 6 3P/FDA diets and 1 non-3P/FDA diet), (3) chicken/turkey (present in 1 3P/FDA diet and 8 non-3P/FDA diets), and (4) rice (present in 0 3P/FDA diets and 7 non-3P/FDA diets). In the current study, potatoes and sweet potatoes were insufficiently represented in either diet type (present in 2 3P/FDA diets and in 1 non-3P/FDA diet) to evaluate.

**Figure 2:**
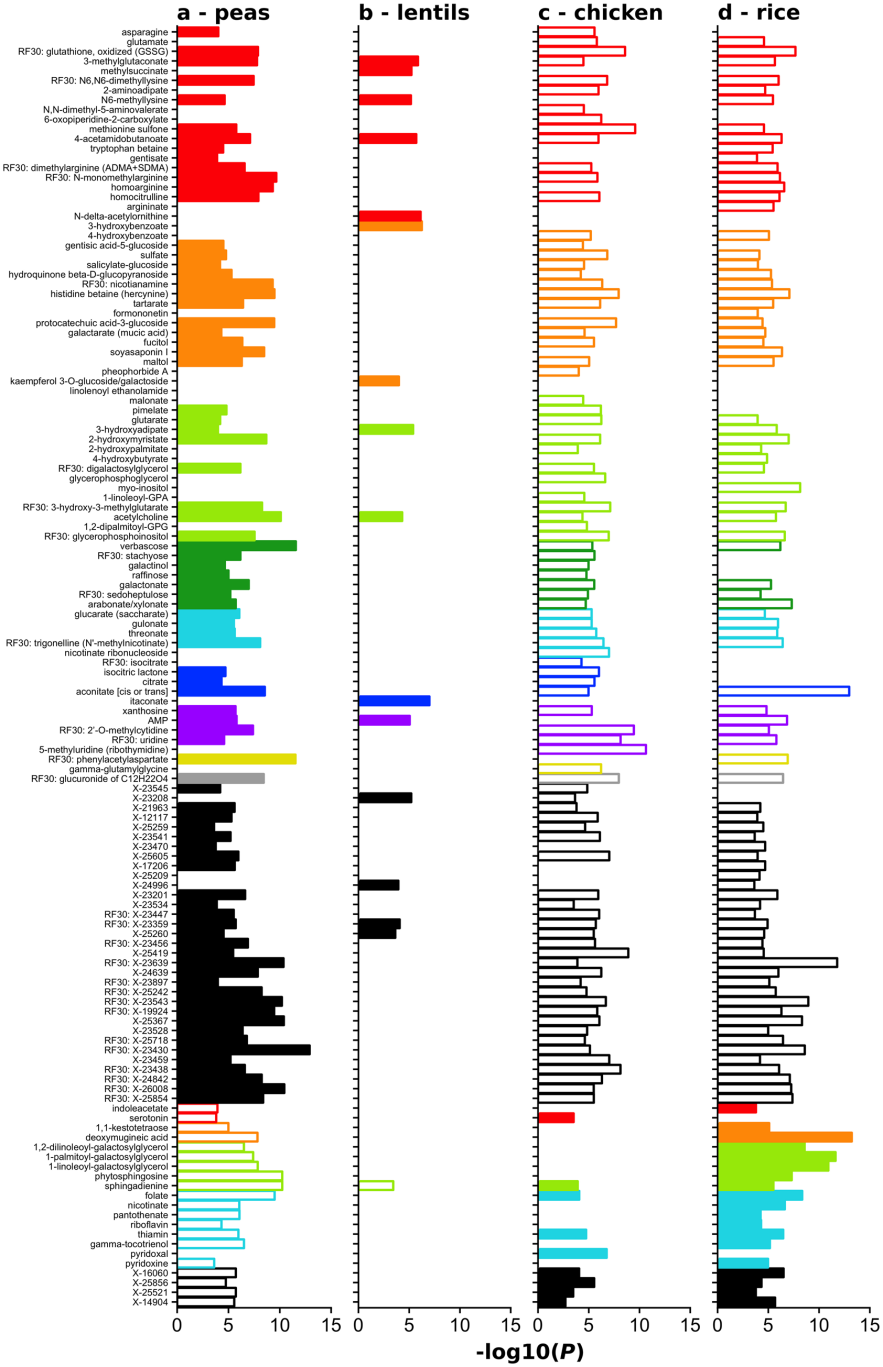
Relationships between four key ingredients and biochemical compounds found to distinguish 3P/FDA from non-3P/FDA diet groups. Panels illustrate the data as follows: (**a**) pea; (**b**) lentil; (**c**) chicken/turkey; (**d**) rice. For each of four ingredients that distinguished 3P/FDA and non-3P/FDA diets (i.e., peas, lentils, chicken/turkey, and rice), the mean level of each compound was compared between diets high versus absent in each ingredient (e.g., diets high in peas compared to diets containing no peas). The *P* value is depicted as − 1*(log10[*P* value]) on the x-axis. Solid (filled) bars indicate a significant positive correlation between compound and ingredient (e.g., diets high in peas had significantly higher levels of a compound compared to diets with no peas), while open bars indicate a significant negative correlation between compound and ingredient. For relationships that do not meet statistical significance, no bar is plotted. Colors represent the classification of the compounds: red, amino acids; orange, xenobiotics and plant-derived compounds; light green, lipid; dark green, carbohydrate; cyan, cofactors and vitamins; blue, energy; violet, nucleotides; yellow, peptide; gray, partially characterized; black, unnamed. The designation RF30 indicates a member of 30 compound set determined by random forest analyses to distinguish the 3P/FDA and non-3P/FDA diet groups.

The relationship between each of these four distinguishing ingredients (peas, lentils, chicken/turkey, and rice) and the biochemical compounds that differed by diet group was visualized by plotting the log2 ratio of the mean concentration of each compound at two ingredient rankings (absent versus high) against the *P* value representing the difference between the two concentrations at the two ingredient rankings (Fig. 2).

Figure 2 illustrates the compounds that are higher and lower in the 3P/FDA diet group compared to the non-3P/FDA diet group. Solid (filled) horizontal bars indicate a positive relationship between an ingredient and a compound. When all four distinguishing ingredients are plotted (Fig. 2), the ingredient-compound relationship for peas shows more solid bars for peas compared to the other ingredients, supporting the possibility that peas contribute to higher concentrations of these biochemical compounds. In contrast to peas, rice and chicken/turkey are associated primarily with open bars, indicating lower amounts of the compounds in association with these ingredients. Compared to peas, ingredient-compound associations for lentils are fewer, but in a similar direction as peas.

The last 21 compounds of Fig. 2 (beginning with indolacetate) illustrate the compounds that are lower (open bars) in the 3P/FDA diet group compared to the non-3P/FDA diet group and their relationships to the same four ingredients. Peas were associated with significantly negative ratios (i.e., lower concentrations of the compound; open bars) for 20 compounds. Lentils are associated with lower concentrations of one compound that is similarly associated with peas. Chicken/turkey and rice are both positively associated with compounds (9 and 19, respectively) that are lower in the 3P/FDA diet group.

These results suggest that peas represent the ingredient contributing the greatest differences between 3P/FDA and non-3P/FDA diets, and that they are associated with higher concentrations of many compounds (88 named biochemical compounds were significantly higher in the 3P/FDA group and 23 named biochemical compounds that were significantly lower in the 3P/FDA group). Generally, compounds that were higher in the high pea diets are lower in the high chicken/turkey or high rice diets.

## Discussion

The current study identified a substantial number of biochemical differences in the 3P/FDA diets, which have been associated with DCM in pet dogs, compared to non-3P/FDA diets^34,40–46^. Most (81.9%) biochemical compounds that differed between diet groups were higher in 3P/FDA diets compared to non-3P/FDA diets, and four distinguishing ingredients, peas, lentils, chicken/turkey, and rice, which also differed across the diet groups, appear to contribute to these differences. Peas and, to a lesser degree, lentils appear to be a possible primary source for the biochemical compounds found to be significantly higher in 3P/FDA diets. While we cannot establish with certainty if any of these compounds and ingredients are causal for disease, the findings support peas as a leading possible ingredient associated with diet-associated DCM in dogs. In general, diets containing peas were commonly associated with compounds being higher in the 3P/FDA diets, rather than with compounds that were lower or deficient in these diets.

One possible hypothesis regarding how the 3P/FDA diets may contribute to DCM posits that these diets could be insufficient in key nutrients that lead to disease. With respect to nutrient insufficiency, several compounds that are relevant to cardiac metabolism, including B vitamins and related compounds, were lower in 3P/FDA diets. B vitamins are co-factors in numerous reactions relevant to cardiac metabolism. Because vitamins B6 and B12, for example, are co-factors in carnitine and taurine synthesis (both of which are important for normal myocardial function), a deficiency or insufficiency in B vitamins potentially could contribute to DCM^52,53^. B vitamins are heat-sensitive, so the results could be related to the amount of B vitamins included in the formulations by individual manufacturers and to levels remaining after extrusion. For example, a study of canned cat foods that were all labeled as complete and balanced found that 12/90 of the diets were below the Association of American Feed Control Officials minimums for thiamine. Pre- and post-manufacture storage conditions also could affect B vitamin levels^54^. Taurine and carnitine, however, were not significantly different between the diet groups. Thiamine (vitamin B1) deficiency also can cause DCM directly^12,55^. Investigation of potential mechanisms related to B vitamins will require additional analyses, including metabolomics studies in dogs with diet-associated DCM.

In contrast to the small number of compounds that were significantly lower in the 3P/FDA diet group, a large set of biochemical compounds were higher. Within the set of compounds that was significantly higher, the categories of amino acids, betaines, and xenobiotics/plant-based compounds are well represented. Although nutrient deficiencies are more obviously suggested when dietary nutrients are lower, such as the B vitamins noted above, deficiencies also can be created indirectly by excess or unbalanced food components that interfere with the normal absorption of a nutrient in the gastrointestinal tract. For example, dietary fiber could alter the digestibility and bioavailability of essential nutrients that appear to be adequately supplied in the diet, contributing to a deficiency^56^. A particular nutrient also could appear to be adequate in the diet but it may not be bioavailable to the animal in a particular form (e.g., organic vs. non-organic sources of zinc, copper oxide vs. copper sulfate)^57,58^. Anti-nutritional factors naturally present in certain ingredients also could theoretically play a role, although most should be inactivated by heat during the manufacturing process.

In the current study, the over-represented categories of amino-acid related compounds, betaines, and xenobiotics/plant-based compounds also could be contributing to deficiencies in molecules that are essential to heart function, such as carnitine. Carnitine is critical in cardiac metabolism through its facilitation of the transport of long chain fatty acids from the cytosol and across the mitochondrial inner membrane for beta-oxidation. Carnitine itself did not differ across the two diet groups, but other amino acid-related compounds that were higher in the 3P/FDA diets could influence carnitine metabolism. Specifically, several compounds that were previously shown to alter carnitine transport could affect carnitine bioavailability to cardiac tissue. The high affinity carnitine transporter, primarily designated as SLC22A5 and formerly called OCTN2 (organic cation transporter novel 2), was identified and characterized over two decades ago^59^, and its connection to DCM described over the last decade^60^. Grube et al. noted that OCTN2 expression in heart tissue was lower in human patients with DCM, and that genetic variants of *SLC22A5* caused heart failure through reduced uptake of carnitine. Several compounds found to be higher in 3P/FDA diets in the current study have been demonstrated to reduce L-[3H] carnitine transport into the myocardium through inhibition of the transporter^59,61^. These inhibitors include the D isomer of carnitine, acetyl-D,L carnitine, gamma butyrobetaine (deoxycarnitine), choline, and betaine^59,61^, and the first two of these carnitine transport inhibitors were higher in 3P/FDA diets. A third compound, 5-aminovaleric acid betaine (N,N,N-trimethyl-5-aminovalerate) was shown to inhibit beta-oxidation of fatty acids in mouse cardiomyocytes, also through inhibition of the carnitine transporter^40^ and was also higher in 3P/FDA diets in the current study. These findings support the possibility that 3P/FDA diets supply biochemical compounds that limit carnitine bioavailability at the level of the mitochondria, interfering with fatty acid oxidation and reducing the heart’s energy supply. Altogether, these relationships support one potential means by which 3P/FDA diets contribute to DCM, although others are possible through various mechanisms that affect myocardial metabolism.

In addition to deficiencies, other possibilities through which the 3P/FDA diets could cause DCM are compounds that have direct toxic effect on the myocardium. For example, plants such as almonds, fruits, and beans can release cyanide, which is cardiotoxic^62^. In the current study, a large number of “unnamed” compounds differed between 3P/FDA and non-3P/FDA diets. Additional work is needed to identify them and any potential role they may play in heart function. We are also unable to exclude the possibility that added or naturally occurring chemicals (e.g., pesticides, mycotoxins, and heavy metals) are present as toxic contaminants in the foods, but were not detected through metabolomics.

The current project focuses on small molecules in dog foods that have been associated with DCM, but at least two relevant studies have assessed metabolites in samples of humans with and without DCM^63,64^. Because many compounds are derived from foods and could affect disease risk without undergoing metabolic conversion, findings from these previous studies are relevant to understanding diet-disease relationships. In these published reports, six compounds were significantly higher in human DCM patients^63^ or in humans with increased disease severity^64^: glutamic acid, 3-hydroxymethylglutaric acid, myo-inositol, 4-acetamidobutanoate, kynurenine, and gamma-glutamylisoleucine. In the current study, all six of these compounds also were significantly higher in the 3P/FDA diets compared to the non-3P/FDA diets. In contrast, higher levels of three compounds—3-hydroxyisovaleric acid (also called beta-hydroxyisovalerate), aminoadipic acid, and citric acid—were associated with protection against DCM in human patients^64^. These three compounds were all higher in 3P/FDA (DCM-associated) diets in the current study. These findings reinforce the need to follow up this foods-based research in dogs and humans, including feeding studies in dogs with and without DCM, to understand the relationships between foods, biochemical compounds, and heart disease in both species.

Although the primary objective of this metabolomics approach was to identify biochemical compounds that distinguished 3P/FDA and non-3P/FDA diets, the impact of ingredients in contributing to these differences was also of interest. Peas emerged as one ingredient that differed across the two diet groups, and was also positively and strongly associated with many compounds that were higher in 3P/FDA foods. Several independent studies, including dietary interventions that used metabolomics analyses, previously confirmed associations between peas and several compounds (trigonelline, dimethylglycine, and tryptophan betaine) that our study identified as higher in the 3P/FDA diets^65–67^. Although trigonelline (nicotinic acid N-methylbetaine) can be classified as a product of niacin (vitamin B3) metabolism in mammals, it also occurs in many plants^67^. Trigonelline was identified as a marker of pea intake in a human feeding study^65^. A second, randomized, crossover study in people identified tryptophan betaine, trigonelline, and dimethylglycine as markers of legume intake^66^. It is unclear whether any of these or other compounds found in high levels in peas could have negative effects on the heart when fed in large amounts, but this warrants further investigation. Other compounds that distinguished 3P/FDA and non-3P/FDA diet groups, such as the tetrasaccharide stachyose, are found in legumes but are not specific to peas^68,69^. Previous reports on ingredients that contribute to DCM in dogs have implicated pulses in general or specific pulses such as lentils or peas^34,35,43,44^. As of April 30, 2019, 89% of DCM-associated diets reported to the FDA contained peas and 93% of diets contained peas or lentils^43^. In the current study, lentils were not as strongly associated with compounds that were significantly different between diet groups although their patterns were similar to those of peas. Our results do not support a role for other pulses (e.g., chickpeas), which were present much less frequently than peas in the diets we analyzed, but additional research is warranted to fully evaluate the associations with pulses other than peas.

Numerous limitations must be acknowledged. While 18 dog food products were analyzed (nine of each diet group), the selection was based on diets that were associated with clinical cases of DCM seen by the authors. All diets in the 3P/FDA group contained ≥ 3 pulses, potatoes, or sweet potatoes in the top 20 ingredients and all were in the top 16 dog food brands named most frequently in DCM cases reported to the FDA^43^. However, it is important to note that there was some subjectivity in the selection and the diets in both groups represent a small number of products relative to all dog foods available in the US marketplace. Although informative, the individual selections and even the diet categories may not be the optimal categorization and additional research will continue to add to the knowledge base to refine the design of future studies and clinical recommendations. It is possible that the causative or contributing factor(s) in the 3P/FDA diets was one of the many unnamed compounds identified or a factor was not measured in the current metabolomic analysis. That only two food samples were analyzed from each selected product, and not all samples were obtained from two different bags of food. (i.e., in some cases, both samples were from a single bag) is another limitation. Some of the diet samples were obtained directly from owners of dogs with DCM; most of these diets had been opened and stored for variable periods of time, first by the owner and then by the investigators, so this could introduce variability and could bias the 3P/FDA diets in having lower levels of certain nutrients that could degrade as a result of variable storage conditions. In addition, the relative rank for each ingredient (absent, low, moderate, high) was based on the ingredient’s position on the food label and was not based on quantitative data on the exact amount of peas or other ingredients that are present in each individual product. Finally, all pea fractions (e.g., peas, pea protein, pea starch, pea fiber) were classified in the same way and it is not yet known whether pea fractions have similar effects.

The current study used a metabolomics approach to foods (“foodomics”) to identify molecular and potential ingredient sources that differentiate two diet groups reported to alter DCM risk in pet dogs. Biochemical compounds that differed by diet group included amino-acid related compounds, carnitine-related molecules, the set of methylated compounds referred to as betaines, and a variety of unnamed xenobiotics and plant compounds, and we hypothesize that one or more of these compounds may contribute to DCM in dogs. The hypotheses generated will require follow-up interventions with metabolomics in dogs to establish causality and provide definitive diet-disease evidence. Finally, while this study focuses on a specific form of heart disease in dogs, it demonstrates the usefulness of investigating relationships between biochemical compounds, food ingredients, and diseases such as DCM.

## Methods

Biochemical analyses were limited to commercial dog foods. The project did not include any interaction with or any experiments on vertebrate animals. All analyses were carried out in accordance with relevant guidelines and regulations including validation by Clinical Laboratory Improvement Amendments (CLIA), ISO 9001certification and accreditation by the College of American Pathologists and the New York State Department of Health Clinical Laboratory.

### Diets

Nine dry (extruded) dog foods that clinically appeared to be associated with DCM in pet dogs were selected for analysis. These nine diets all contained at least three occurrences of pulses, potatoes, or sweet potatoes in the top 20 ingredients and were in the top 16 dog food brands fed to dogs with DCM reported to the FDA in the agency’s update in June, 2019 (3P/FDA diets)^43^. In addition, nine dry dog foods that met World Small Animal Veterinary Association recommendations^70^, were not, in the authors’ experience, associated clinically with DCM in dogs, and did not meet the other criteria for the 3P/FDA diets were selected for analysis as controls (Non-3P/FDA diets). Two samples from each product were obtained so that each product could be tested in duplicate. Where possible, samples that were obtained directly from owners of dogs with DCM were used for the analysis. For these diets, only a single bag of food was used and two samples from separate parts of the bag were selected. Most of these diets had been opened by the owner and stored for variable periods of time, first by the owner and then by the investigators. When diets being fed to individual dogs were not available, two new bags from the same lot were purchased and one sample from each bag was collected for analysis. All samples were labeled with a code so the identity was unknown to the laboratory. Samples were shipped overnight to a commercial laboratory (Metabolon, Inc., Morrisville, NC).

### Metabolomic analysis

Metabolic profiling of two samples from each of 18 dog food products was conducted by Metabolon, Inc. using standardized methods. Biochemical compounds were quantified using ultra‐high‐performance liquid chromatography–tandem mass spectroscopy and identified by comparison to a reference library of 4500 purified standards containing retention time, molecular weight, mass–charge ratio, and mass spectroscopy spectral data. A total of 830 biochemical compounds were detected, identified, and met quality control requirements according to standard protocols of the commercial laboratory. All identified compounds, both named and unnamed, were included in the analyses.

### Ingredient ranking

Diets’ ingredient lists varied both within and between diet groups (i.e., 3P/FDA and non-3P/FDA). While some ingredients were present only in one diet group (for example, grains were present only in the non-3P/FDA diet group), other ingredients were shared across both diet groups. Ingredient positions on dog food labels, like human food labels, indicate relative amounts of each ingredient by weight. To facilitate analyses of the relationships between ingredients and biochemical compounds, a relative rank for each ingredient (absent, low, moderate, high) was assigned by one of the authors (LMF, a veterinary nutritionist) based on the ingredient’s position on the food label.

### Identification of distinguishing ingredients

Dog foods included a variety of ingredients, with up to 57 ingredients in a single diet. When all the different ingredients across the 18 different diets were added, several hundred ingredients were represented, some of which are contained only in a single food or a few foods, while others were common within a diet group. We identified major distinguishing ingredients that differed by five or more dog foods across the two diet groups. For example, rice was present in 7/9 non-3P/FDA diets and 0/9 3P/FDA diets, yielding a difference (7–0) of 7. Mineral supplements and probiotics were excluded from ingredients analysis. Ingredients that differed by five or more dog foods when compared across 3P/FDA and non-3P/FDA diet groups were considered “distinguishing ingredients.”

### Statistical analyses

Linear regression was used to assess associations between the 3P/FDA and non-3P/FDA diet groups and the individual biochemical compounds present in the foods. A conservative Bonferroni correction for 830 biochemical compounds was applied to determine statistical significance (0.05/830 = 0.00006). *P* values < 0.05 and ≥ 0.00006 were considered to be nominally significant.

Random forest classification was applied to biochemical compounds in 3P/FDA and non-3P/FDA diets to identify the top 30 compounds that yielded a predictive accuracy of 100%. These compounds were identified through “importance” rank ordering that was generated through random permutation.

Analysis of the relationship between ingredients and biochemical compounds that differed significantly between 3P/FDA and non-3P/FDA diets was assessed by pair-wise comparison of compounds when each of four distinguishing ingredients was ‘absent’ compared to when each ingredient was ranked ‘high’. Statistical significance for ingredient-compounds analyses was conservatively corrected by multiplying the number of compounds tested by the number of ingredients tested (four). Specifically, for named compounds that were higher in 3P/FDA foods (n = 88 compounds) the threshold P threshold was calculated as 0.05/(88 × 4) to yield *P* = 0.0001. For named compounds that were lower in 3P/FDA foods (n = 23), the P threshold was calculated as 0.05/(23 × 4) to yield *P* = 0.0005. Similar methods were applied for unnamed compounds. For unnamed compounds that were higher in 3P/FDA foods (n = 34), the threshold *P* value was 0.0004 and for unnamed compounds that were lower in 3P/FDA foods (n = 4), the threshold *P* value was 0.003. Statistical analyses for biochemical compounds and ingredients were conducted in SAS 9.4.

## Supplementary Information

Supplementary Information.

## Data Availability

The datasets generated during and/or analyzed during the current study are not publicly available due to ongoing research but are available from the corresponding author on reasonable request.
